# 10-Year Change in the Laboratory-Based Prevalence of Chronic Kidney Disease in Patients from a Brazilian Cardiologic Center

**DOI:** 10.3390/epidemiologia7030087

**Published:** 2026-06-22

**Authors:** Farid Samaan, Rubens Carvalho Silveira, Kleber Gomes Franchini, Fausto Feres, Gianna Mastroianni-Kirsztajn, Ricardo Sesso

**Affiliations:** 1 Dante Pazzanese Institute of Cardiology, São Paulo 04012-909, SP, Brazil; kleber.franchini@dantepazzanese.org.br (K.G.F.); fferes@dantepazzanese.org.br (F.F.); 2 State Department of Health of São Paulo, São Paulo 04039-000, SP, Brazil; 3 School of Public Health, University of São Paulo, São Paulo 01246-904, SP, Brazil; rubenscsilveira@gmail.com; 4 Department of Medicine (Division of Nephrology), Federal University of São Paulo, São Paulo 04039-000, SP, Brazil; gm.kirsztajn@unifesp.br (G.M.-K.); rsesso@unifesp.br (R.S.)

**Keywords:** epidemiology, chronic kidney disease, cardiovascular disease, cardiac care facilities, creatinine, proteinuria

## Abstract

**Background:** We aim to estimate the variation in the prevalence of chronic kidney disease (CKD) in patients from a Brazilian cardiologic center. **Methods:** The outpatient serum creatinine level and urine albumin–creatinine ratio (UACR) in samples from patients ≥18 years old between 2014 and 2023 were evaluated. CKD was defined as an estimated glomerular filtration rate (eGFR) < 60 mL/min/1.73 m^2^. Participants were categorized into low-, moderate-, high- or very high-risk groups according to the CKD heatmap, which combines eGFR with UACR results. **Results:** The mean number of adults with serum creatinine results per year was 36,477 ± 7239, and the mean number of those with UACR results was 16,870 ± 4310. The age- and sex-adjusted prevalence of participants with CKD increased significantly (from 20% to 31%; R^2^ = 0.853; *p* < 0.001), as was the prevalence of individuals in the high or very high CKD risk groups (14% to 21%; R^2^ = 0.945; *p* < 0.001). The cumulative incidence of CKD during the study period was 21.7% and was higher in females and in older age groups. **Conclusions:** The roughly 50% increase in the laboratory-based CKD prevalence over 10 years underscores the need for healthcare services to adapt to managing a population with growing complexity and a heightened risk of requiring kidney replacement therapy.

## 1. Introduction

The prevalence of individuals receiving kidney replacement therapy (KRT) has increased exponentially worldwide [1]. This is explained by the increased prevalence of risk factors for chronic kidney disease (CKD), increased life expectancy, and increased availability of KRT [2]. The incidence of people on dialysis is also rising, especially in developing countries and those with pronounced demographic and epidemiological transitions (aging population and increase in the prevalence of chronic noncommunicable diseases) [3].

Among these nations, Brazil stands out as an upper-middle-income Latin American country that has the fourth-largest chronic dialysis population (behind the United States, China and Japan) and among the ten countries with the greatest increases in the prevalence of individuals on haemodialysis or peritoneal dialysis [4]. In addition, the prevalence of the main risk factors for CKD has increased in Brazil. Between 2000 and 2020, the percentage of people aged over 60 years increased from 9% to 13% and the percentage of adults with arterial hypertension, diabetes, and obesity increased from 21% to 25%, 5% to 9%, and 11% to 23%, respectively [5,6].

International studies, especially in populations of high-income countries, have shown that the prevalence of CKD in adults is 10–15% [7,8]. In Brazil, recent studies have shown a prevalence of CKD of 4% in individuals with private health plans, 13% in users of private laboratories and 31% in patients with heart disease [9,10]. Although the prevalence of patients with risk factors for CKD is known to have increased [11,12], data on the epidemiological trends of CKD in people who are not on dialysis are scarce.

Among the chronic diseases associated with CKD, cardiovascular disease (CVD) has drawn significant attention because CKD and CVD share many risk factors, the prevalence of both conditions is increasing, and CKD greatly worsens the prognosis of CVD patients and vice versa [13].

The COVID-19 pandemic had a significant impact on the provision of preventive care in Brazil, as it did in other non-high-income countries [14]. Some procedures related to CKD care showed a recovery after 2021, such as consultations with nephrologists and testing for serum creatinine and proteinuria, while others, such as kidney transplants, had not returned to pre-pandemic rates as of December 2024 [15].

As few epidemiological studies on kidney and heart disease have been conducted in non-high-income countries, we aim to estimate the variation in the laboratory-based prevalence of chronic kidney disease (CKD) in patients from a Brazilian cardiologic center.

## 2. Materials and Methods

### 2.1. Study Design and Population

This 10-year cross-sectional analysis utilized data from the laboratory databases of a public tertiary hospital specializing in cardiology located in the city of São Paulo, Brazil. This hospital is a reference hospital for a population of 20 million inhabitants; has 360 beds; and performs approximately 189,000 outpatient medical consultations, 22,000 emergency medical consultations, 9000 haemodynamic procedures and 3500 cardiac surgeries annually [16]. The laboratory tests evaluated in this study were performed between 1 January 2014 and 31 December 2023, which were mainly requested by cardiologists. In our institution, a small proportion of patients (approximately 3–5%) are treated by nephrologists, ophthalmologists, and endocrinologists in specialized outpatient clinics. The inclusion criteria were as follows: age ≥ 18 years and tests performed on an outpatient basis. For patients with two or more tests in the same year, the worst values were excluded to prevent overestimation of the CKD prevalence.

### 2.2. Data Collection and Definitions

The tests evaluated in this study were serum creatinine (sCR) level and the albumin–creatinine ratio in isolated urine samples (UACR). In addition to these test results, the following information was collected: age and sex of the patient and date of sample collection. Using the sCR level, age and sex, the estimated glomerular filtration rate (eGFR) was calculated using the CKD-EPI 2021 equation [17]. CKD was defined as an eGFR < 60 mL/min/1.73 m^2^, and proteinuria was defined as a UACR > 30 mg/g. CKD was classified as stage 3a (eGFR 45–59 mL/min/1.73 m^2^), stage 3b (eGFR 30–44 mL/min/1.73 m^2^), stage 4 (eGFR 15–29 mL/min/1.73 m^2^) and stage 5 (eGFR < 15 mL/min/1.73 m^2^) [18]. Proteinuria was classified as mild (UACR < 30 mg/g), moderate (UACR 30–300 mg/g) or severe (UACR > 300 mg/g) [18]. Patients with sCR and UACR results measured in the same year were classified according to the Kidney Disease: Improving Global Outcomes (KDIGO) heatmap into the following CKD risk categories: low, moderate, high and very high [18].

### 2.3. Study Outcomes

The primary outcomes of the study were the annual age- and sex-adjusted prevalence of patients with CKD (eGFR < 60 mL/min/1.73 m^2^), severe proteinuria (UACR > 300 mg/g) and high or very high CKD risk categories.

The secondary outcome of the study was the cumulative incidence of CKD. To estimate the CKD incidence, a subsample of individuals with three or more sCR measurements was used, where the first two tests corresponded to a preserved eGFR (>60 mL/min/1.73 m^2^). From that point on, participants were followed over time to identify the moment when they presented an eGFR ≤ 60 mL/min/1.73 m^2^. When this occurred, the individual was considered an incident case, and the time to the change in test results was recorded. The incidence was then estimated for each follow-up period, and subsequently, these incidences were accumulated over time, resulting in the estimate of the cumulative incidence.

### 2.4. Statistical Analysis

Categorical variables are described as absolute and relative frequencies, and continuous variables are described as median (interquartile). Crude prevalence calculations (number of individuals with abnormal results in the total number of those who underwent the exam) were performed annually and adjusted for age and sex. The standard population for the adjustments was calculated by the direct method, taking the mean distributions of age and sex over the 10 years of the study into account. The annual variation in prevalence was calculated via Prais–Winsten regression [19]. The time between the first measurement of sCR with eGFR > 60 mL/min/1.73 m^2^ and the first measurement of sCR with eGFR < 60 mL/min/1.73 m^2^ in the same individual was used to calculate incidence (new cases of CKD per year). Statistical analysis was performed using R software, version 4.2.1, using the following packages: nephro, tidyverse, abjutils, lubridate and prais.

## 3. Results

### 3.1. CKD Prevalence

The number of participants with sCR measurements each year ranged between 21,228 and 45,112. These cases account for about 15 to 25 percent of the people who had medical outpatient consultations at the study site [16]. The percentage of males with sCR measurements varied from 50.0% to 51.8% and the median (IQR) age ranged between 64.6 (56.1;72.9) and 67.5 (58.7;75.1) years. The crude prevalence of CKD over the years evaluated was: 20.0% (2014), 21.2% (2015), 19.2% (2016), 19.7% (2017), 24.5% (2018), 23.2% (2019), 27.1% (2020), 24.5% (2021), 28.8% (2022), and 30.7% (2023) (Table 1).

The number of participants with simultaneous sCR and UACR measurements varied from 8813 to 21,511. The frequency of UACR measurements ranged between 24.3% and 54.9%. The percentage of males varied from 47.8% to 50.8%. and the median (IQR) age ranged between 65.0 (58.5; 73.7) and 68.1 (59.6; 75.3) years. The percentage of participants with the high or very-high CKD risk category varied from 14.6% to 20.4% (Table 2).

**Figure 1:**
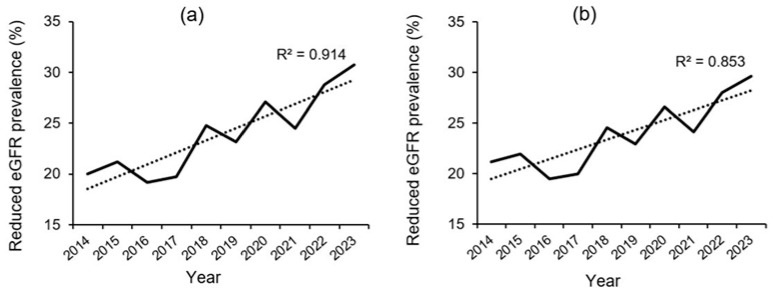
Variation in reduced eGFR prevalence (<60 mL/min/1.73 m^2^) over 10 years. (**a**) Crude prevalence; (**b**) age- and sex-adjusted prevalence.

Between 2014 and 2023, the crude prevalence of adults with eGFR < 60 mL/min/1.73 m^2^ ranged between 20.0% and 30.7%, with a significant increase over time (R^2^ = 0.914; *p* < 0.001). After adjusting for age and sex, the increase was still significant (21.2–29.6%; R^2^ = 0.853; *p* < 0.001) (Figure 1).

**Figure 2:**
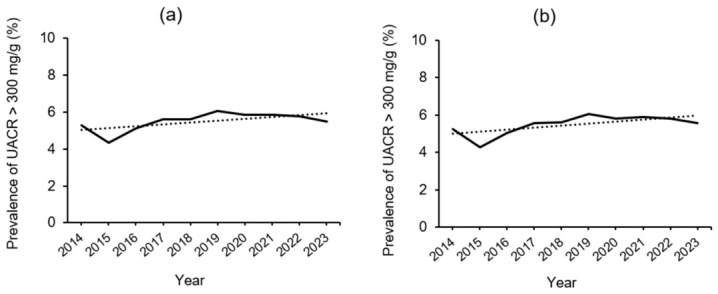
Variation in the prevalence of severe proteinuria (urinary albumin/creatinine ratio [UACR] > 300 mg/g) over 10 years. (**a**) Crude prevalence; (**b**) age- and sex-adjusted prevalence.

Between 2014 and 2023, the crude prevalence of adults with UACR > 300 mg/g increased slightly but not significantly (4.3–6.1%; R^2^ = 0.317; *p* = 0.053), as did the annual prevalence adjusted for age and sex (4.3–6.1%; R^2^ = 0.317; *p* = 0.052) (Figure 2).

**Figure 3:**
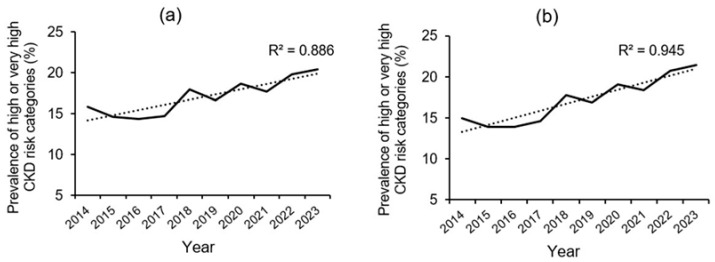
Variation in the prevalence of the high- or very high-risk categories of chronic kidney disease (CKD) over 10 years. (**a**) Crude prevalence; (**b**) age- and sex-adjusted prevalence.

Between 2014 and 2023, the annual crude prevalence of adults in the high or very high CKD risk categories ranged between 14.6% and 20.4%, with a significant increase over time (R^2^ = 0.886; *p* < 0.001). After adjusting for age and sex, the increase was still significant (13.9–21.4%; R^2^ = 0.945; *p* < 0.001) (Figure 3).

### 3.2. CKD Incidence

**Figure 4:**
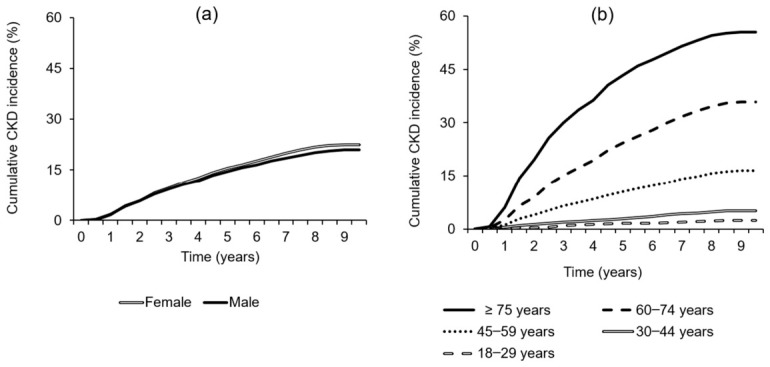
Cumulative incidence of reduced eGFR (<60 mL/min/1.73 m^2^) according to sex (**a**) and age group (**b**).

The proportion of new patients with one or more sCR measurements each year ranged from 15.6% to 33.2% (Table S1). The total number of participants at risk for CKD (i.e., adults with at least two eGFR above 60 mL/min/1.73 m^2^) was 39,414 and the 10-year incident cases of CKD (one or more eGFR < 60 mL/min/1.73 m^2^) were 8547 (21.7%) (Tables S2 and S3). The cumulative incidence of CKD (eGFR < 60 mL/min/1.73 m^2^) over the 10 years of the study was greater in females than in males (22.5% versus 20.9%; *p* < 0.001). The cumulative incidence of CKD was greater in older participants (2.4%, 5.1%, 16.5%, 35.8% and 55.5% in the >18 years, 30–44 years, 45–59 years, 60–74 years and ≥ 75 years age groups, respectively; *p* < 0.001) (Figure 4).

## 4. Discussion

The present study revealed that the prevalence of reduced eGFR significantly increased in patients with heart disease in the last decade (from 20% to 31%), as did the prevalence of patients at high or very high CKD risk categories (from 16% to 20%). The prevalence of individuals with severe proteinuria did not increase significantly. The cumulative incidence of CKD in the total study population was approximately 20% over 10 years, with the incidence being greater in females and older age groups.

Accordingly, to the last Global Burden Disease Study, the age-standardized CKD prevalence in the world regions was: 5 to 11% in Europe and Central Asia, 6 to 8% in high-income countries from Asia, Oceania, and North America, 6 to 9% in Latin America, and 6 to 9% in Africa [20]. Compared to the general population, this and other studies have shown a higher prevalence of CKD in people with heart disease. While the crude prevalence of CKD among all adults is estimated at 10–15% [7,8,10], the condition is present in 21% of individuals with coronary heart disease, 32% of those with heart failure and 37% of those with valvular heart disease [9,21,22]. Although the comorbidities of all the study participants were not available, a previous report showed that among the patients treated at this institution, 32% had coronary artery disease, 14% had valvular heart disease, 10% had arrhythmias, and 9% had cardiomyopathies [9]. The high coexistence of CVD and CKD is mainly attributed to older age and a higher prevalence of hypertension and diabetes mellitus. In addition, non-traditional risk factors for CVD may be present in individuals with CKD, such as bone mineral disease/vascular calcification, inflammation, oxidative stress, endothelial dysfunction, hyperuricemia, and hypervolemia [18].

It is well known that the prevalence of people on dialysis is increasing worldwide; nevertheless, the variation in the prevalence of CKD has been less often studied, especially in low- and middle-income countries [23,24]. One hypothesis for the increased prevalence of CKD observed in our study could be the increased prevalence of risk factors for the disease in Brazil (hypertension, diabetes, obesity and the ageing population) [5,6]. In the United States, the prevalence of patients with a reduced eGFR (below 60 mL/min/1.73 m^2^) increased 43% between national surveys from 1988–1994 and 1999–2002 [25]. During this period, the prevalence of hypertension, diabetes and obesity increased from 24% to 26%, from 4% to 7% and from 23% to 30%, respectively [26]. On the other hand, a Norwegian report showed that the prevalence of CKD remained stable between the population studies of 1995–1997 and 2006–2008 [26]. The authors estimated that the reduction in blood pressure and cholesterol levels, stabilization of the prevalence of diabetes and increase in physical activity of the population prevented an increase of up to 3% in the prevalence of CKD in that country [26].

The trend toward an increase in the prevalence of individuals with reduced eGFR remained statistically significant after adjusting for age and sex. The decrease in the coefficient of determination (R^2^) from 0.914 to 0.853 and the 18.8% decrease in the slope of the curve confirm the influence of demographic characteristics on the study results.

While a sudden increase in the crude prevalence of CKD was observed in 2020—possibly related to the greater severity of patients treated on an outpatient basis during the COVID-19 pandemic—the upward trend continued in the subsequent years evaluated. In fact, even after excluding year 2020 from the analyses, the increasing tendency in the crude prevalence and the age- and sex-adjusted prevalence of CKD remained statistically significant (R^2^ = 0.711; *p* = 0.002 and R^2^ = 0.600; *p* = 0.006, respectively) (Figure S1).

The increase in the prevalence of CKD and its risk factors are not the only determinants of the number of people on KRT [23]. Even in nations without restricted access to KRT, the variation in the prevalence of CKD explains only 40% of the increase in the prevalence of individuals on dialysis, suggesting that there may be different risk factors for patients with CKD progressing to kidney failure [24]. Thus, the fact that Brazil is one of the ten countries in the world with the greatest incidence of patients on dialysis could be explained by both the increased prevalence of CKD and its risk factors and the gaps in coping with the disease, which increase the risk of end-stage kidney disease [27,28].

The increase in the CKD prevalence is expected to be accompanied by an increase in the occurrence of proteinuria. However, the findings of this and other studies revealed some stabilization of the prevalence of proteinuria over time [29,30]. One hypothesis to explain this result could be the increased use of medications with antiproteinuric properties that are freely provided by the Government in Brazil (angiotensin-converting enzyme inhibitors and angiotensin receptor blockers), since the study site is a teaching hospital and therefore more likely to adhere to clinical protocols and therapeutic guidelines. Unfortunately, information on the use of medications and comorbidities of the participants in this study was not available. Therefore, in this study, the significant increase in the prevalence of patients in the high- and very-high-risk categories for CKD occurred predominantly due to the increase in the prevalence of individuals with reduced eGFR.

Although Brazilian and international studies have reported the prevalence of CKD [9,10,31,32], analyses of the disease’s incidence are scarce and primarily focus on patients on maintenance dialysis [33]. In addition, several methodological issues make comparisons between studies on the incidence of CKD challenging, such as the study’s population, the lack of proteinuria assessments in most participants and the absence of two eGFR measurements taken at least three months apart [34]. The incidence of CKD was 0.5–0.7% in studies conducted in the general population using the KDIGO definition (i.e., two or more measurements of eGFR < 60 mL/min or proteinuria with an interval of at least 3 months between each biomarker measurement) [18,34,35]. In one of these studies, when only a single eGFR measurement < 60 mL/min/1.73 m^2^ was considered as CKD, the incidence rose from 0.5% to 1.2% [35].

The higher incidence of CKD observed in our study is most likely related to the study population (patients from a tertiary care hospital, older age, and higher burden of comorbidities). However, it should be noted that we used only a single eGFR measurement < 60 mL/min/1.73 m^2^ as the definition of CKD. When CKD was considered only in individuals with two or more measurements of reduced eGFR, the cumulative incidence dropped from 22% to 12% (Table S4).

The occurrence of new cases of patients with reduced eGFR in our study was greater in females and older age groups, which is consistent with the results of previous studies [36]. Some reasons that could explain the sex difference in CKD incidence are the greater life expectancy, lower mortality, lower nephron mass and greater chance of testing in females than in males [36,37]. Advanced age is widely recognized as a risk factor for CKD because of the higher prevalence of hypertension and diabetes and the length of exposure of the kidneys to these and other aggressive factors [38].

This study has limitations that must be acknowledged. First, the retrospective design and unavailability of information on comorbidities, baseline cardiac diagnosis and medication use of the patients did not allow us to generate more detailed results regarding temporal changes in eGFR and albuminuria. Second, even though all adults with CVD are at risk for CKD and should therefore be frequently screened for this condition, individuals with sCR and UACR measurements may have a higher disease burden than those who did not undergo these tests. To partially mitigate the potential for overestimating the prevalence of CKD in this study, we excluded tests performed more than once a year, retaining the lowest sCR and UACR values. Third, it was not possible to use two eGFR measurements with an interval of three months or more to better define CKD (according to the KDIGO guidelines) because approximately 75% of the patients underwent this test only once a year. However, the single measurement of sCR, with or without testing for proteinuria, has been the predominant method of defining CKD in epidemiological studies with large numbers of participants [7,8,9,10]. Even after excluding repeated tests and retaining the better (lower) results for sCR and UACR, we cannot completely rule out the inclusion of cases of transient community-acquired acute kidney injury and the overestimation of the prevalence of CKD in our population. Fourth, no CKD marker other than sCR was used to define kidney disease because the UACR was not measured in approximately 55% of the patients. Previous studies have shown that individuals tested for proteinuria are older and have a greater burden of comorbidities than those not tested [10]; thus, the inclusion of patients with UACR measurements could overestimate the prevalence of CKD. Finally, the study was conducted in a single center, i.e., a tertiary referral hospital in cardiology, linked to the Public Health System. Nevertheless, given the large number of patients treated at this medical service, its central location, and easy accessibility in the urban area, it is likely that the results found correspond to what is observed in the population of patients with heart disease in São Paulo during this period.

## 5. Conclusions

This study utilized data from laboratory databases over a decade, including more than 20,000 patients each year. To our knowledge, this study is the first in Brazil to evaluate temporal variations in the prevalence of CKD in patients with heart disease and represents one of the largest international studies on this topic. The specificity of the studied population is relevant because both CKD and CVD, as chronic conditions, are global priorities in the field of public health. The roughly 50% increase in the laboratory-based CKD prevalence over 10 years underscores the need for healthcare services to adapt to managing a population with growing complexity and a heightened risk of requiring KRT.

## Figures and Tables

**Table 1:** Characteristics of the study participants with serum creatinine measurements.

Variable	Year									
	2014	2015	2016	2017	2018	2019	2020	2021	2022	2023
Patients with sCR values, *n*	45,112	45,100	40,500	37,605	21,228	36,278	28,156	37,555	37,664	35,570
Age, years	64.6 (56.1;72.9)	65.1 (56.7;73.2)	65.6 (57.4;73.5)	65.7 (57.5;73.7)	66.2 (58.0;74.1)	66.5 (57.9;74.3)	66.8 (58.4;74.3)	66.9 (58.3;74.3)	67.2 (58.5;74.7)	67.5 (58.7;75.1)
18–29, % (*n*)	1.8 (833)	1.6 (717)	1.4 (587)	1.5 (557)	1.4 (304)	1.5 (557)	1.4 (388)	1.5 (559)	1.5 (567)	1.5 (551)
30–44, % (*n*)	6.6 (2993)	6.3 (2851)	5.7 (2298)	5.6 (2123)	5.1 (1089)	5.6 (2047)	5.3 (1479)	5.5 (2057)	5.4 (2019)	5.4 (1903)
45–59, % (*n*)	27.0 (12,172)	25.7 (11,603)	24.6 (9956)	24.2 (9102)	23.2 (4925)	22.8 (8254)	22.2 (6241)	21.9 (8218)	21.4 (8061)	21.2 (7536)
60–74, % (*n*)	44.8 (20,230)	45.8 (20,634)	47.2 (19,110)	47.4 (17,821)	47.9 (10,159)	47.1 (17,070)	48.0 (13,519)	48.1 (18,050)	47.8 (17,991)	46.7 (16,620)
75 or more, % (*n*)	19.7 (8884)	20.6 (9295)	21.1 (8549)	21.3 (8002)	22.4 (4751)	23.0 (8350)	23.2 (6529)	23.1(8671)	24.0 (9026)	25.2 (8960)
Male sex, % (*n*)	50.0 (22,534)	50.4 (22,750)	51.3 (20,771)	51.7 (19,449)	51.1 (10,852)	51.1 (18,535)	51.8 (14,585)	51.5 (19,338)	51.6 (19,442)	51.3 (18,253)
eGFR, mL/min/1.73 m^2^	81.4 (64.2;97.9)	80.6 (63.3;97.3)	83.7 (65.4;100.1)	82.4 (64.6;98.8)	77.4 (60.2;94.2)	79.3 (61.6;96.1)	75.0 (58.3; 92.1)	77.4 (60.4;94.0)	73.3 (57.4;88.8)	72.0 (55.9;87.9)
≥60, % (*n*)	80.0 (36,085)	78.8 (35,535)	80.8 (32,738)	80.3 (30,182)	75.5 (15,967)	76.8 (27,873)	72.9 (20,523)	75.5 (28,340)	71.2 (26,833)	69.3 (24,638)
45–59, % (*n*)	12.5 (5635)	13.0 (5848)	12.0 (4843)	11.9 (4479)	14.9 (3159)	13.7 (4984)	15.9 (4481)	14.9 (5599)	17.3 (6526)	17.6 (6268)
30–44, % (*n*)	5.3 (2378)	5.7 (2573)	4.9 (1973)	5.3 (1986)	7.0 (1486)	6.2 (2245)	7.4 (2096)	6.6 (2478)	7.8 (2926)	8.9 (3151)
15–29, % (*n*)	1.4 (616)	1.5 (680)	1.5 (598)	1.5 (546)	2.2 (456)	1.6 (584)	2.0 (571)	1.7 (636)	2.0 (739)	2.2 (785)
<15, % (*n*)	0.9 (398)	1.0 (464)	0.9 (348)	1.1 (412)	0.8 (160)	1.6 (592)	1.7 (485)	1.3 (502)	1.7 (640)	2.0 (729)

Data are presented as medians (IQRs) or relative (%) and absolute (*n*) frequencies. sCR, serum creatinine. eGFR, estimated glomerular filtration rate.

**Table 2:** Characteristics of the study participants with simultaneous serum creatinine and UACR measurements.

Variable	Year									
	2014	2015	2016	2017	2018	2019	2020	2021	2022	2023
Patients with sCR and UACR, % (*n*)	40.6 (18,318)	47.7 (21,511)	52.1 (21,117)	49.3 (18,523)	50.1 (10,640)	24.3 (8813)	52.5 (14,787)	54.9 (20,633)	44.5 (16,761)	49.5 (17,596)
Age, years	65.0 (58.5; 73.7)	66.4 (58.8; 73.9)	66.5 (59.2; 73.8)	66.6 (59.3; 74.0)	67.3 (60.1; 74.6)	67.4 (59.9; 74.5)	67.7 (60.1; 74.9)	67.5 (59.6; 74.7)	68.0 (59.9; 75.2)	68.1 (59.6; 75.3)
18–29, % (*n*)	0.6 (109)	0.7 (157)	0.7 (155)	0.8 (157)	0.7 (74)	0.9 (77)	1.0 (143)	1.3 (277)	1.6 (265)	1.5 (266)
30–44, % (*n*)	5.1 (932)	5.4 (1158)	5.4 (1134)	5.9 (1086)	5.8 (612)	6.8 (599)	6.9 (1019)	7.7 (1592)	8.2 (1376)	8.6 (1508)
45–59, % (*n*)	31.5 (5775)	31.7 (6813)	32.9 (6856)	34.6 (6414)	34.5 (3676)	35.6 (3136)	36.0 (5323)	36.5 (7540)	36.4 (6097)	36.6 (6437)
60–74, % (*n*)	47.2 (8646)	47.4 (10,194)	48.1 (10,157)	47.0 (8706)	47.1 (5011)	45.8 (4036)	45.9 (6790)	44.3 (9146)	44.1 (7387)	43.4 (7628)
≥75, % (*n*)	15.6 (2856)	14.8 (3189)	12.9 (2715)	11.7 (2160)	11.9 (1267)	10.9 (965)	10.2 (1512)	10.1 (2078)	9.8 (1636)	10.0 (1757)
Male sex, % (*n*)	8763 (47.3)	10,497 (48.8)	10,593 (50.2)	9314 (50.3)	5308 (49.9)	4366 (49.5)	7381 (49.9)	10,356 (50.2)	8499 (50.7)	8673 (49.3)
UACR, mg/g	14.4 (6.9; 37.5)	8.0 (4.0; 24.7)	10.1 (4.7; 35.8)	9.3 (4.6; 29.3)	10.0 (4.7; 32.5)	10.2 (4.8; 32.0)	9.9 (4.7; 31.2)	12.7 (6.2; 36.5)	10.8 (5.0; 34.2)	9.5 (4.6; 29.8)
<30, % (*n*)	70.2 (12,856)	77.9 (16,758)	72.2 (15,240)	75.3 (13,941)	73.6 (7834)	73.6 (6487)	74.3 (10,990)	71.4 (14,737)	72.9 (12,211)	75.0 (13,193)
30–300, % (*n*)	24.6 (4498)	17.8 (3823)	22.7 (4801)	19.1 (3546)	20.8 (2210)	20.3 (1793)	19.8 (2934)	22.7 (4688)	21.4 (3587)	19.5 (3438)
>300, % (*n*)	5.3 (964)	4.3 (930)	5.1 (1076)	5.6 (1036)	5.6 (596)	6.1 (533)	5.8 (863)	5.9 (1208)	5.8 (963)	5.5 (965)
CKD risk category										
Low, % (*n*)	57.8 (10,584)	63.5 (13,656)	60.8 (12,832)	62.8 (11,639)	58.0 (6173)	59.8 (5271)	57.8 (8544)	57.2 (11,797)	55.6 (9311)	55.6 (9774)
Moderate, % (*n*)	26.4 (4833)	21.9 (4711)	24.9 (5263)	22.5 (4167)	24.1 (2562)	23.5 (2075)	23.6 (3489)	25.1 (5186)	24.7 (4135)	24.1 (4235)
High, % (*n*)	9.5 (1743)	9.0 (1945)	8.8 (1862)	9.0 (1659)	10.6 (1132)	9.8 (860)	11.1 (1644)	10.7 (2202)	11.8 (1970)	12.2 (2144)
Very high, % (*n*)	6.3 (1158)	5.6 (1199)	5.5 (1160)	5.7 (1058)	7.3 (773)	6.9 (607)	7.5 (1110)	7.0 (1448)	8.0 (1345)	8.2 (1443)

Data are presented as medians (IQRs) or relative (%) and absolute (*n*) frequencies. sCR, serum creatinine. UACR, albumin/creatinine ratio in an isolated urine sample. CKD, chronic kidney disease.

## Data Availability

The data supporting this study is available from the corresponding author upon reasonable request and after authorization from the local research ethics committees.

## Supplementary Materials

The following supporting information can be downloaded at: https://www.mdpi.com/article/10.3390/epidemiologia7030087/s1, Table S1: Number and proportion of new patients with serum creatinine measurements each year; Table S2: Incidence of CKD according to sex; Table S3: Incidence of CKD according to age group; Table S4: Cumulative incidence of CKD according to the methodology adopted for defining new cases; Figure S1: Trends in the prevalence of reduced eGFR (<60 mL/min/1.73 m^2^), excluding the year 2020 (COVID-19 pandemic). (a) Crude prevalence; (b) age- and sex-adjusted prevalence.

## Abbreviations

The following abbreviations are used in this manuscript:

CKD	Chronic kidney disease
CVD	Cardiovascular disease
eGFR	Estimated glomerular filtration rate
KDIGO	Kidney disease: improving global outcomes
KRT	Kidney replacement therapy
sCR	Serum creatinine
UACR	Urine albumin–creatinine ratio
