# Supplementary material for: 10-Year Change in the Laboratory-Based Prevalence of Chronic Kidney Disease in Patients from a Brazilian Cardiologic Center
Source: Epidemiologia (Basel). 2026 Jun 22;7(3):87. doi: 10.3390/epidemiologia7030087 (PMC13297813; doi:10.3390/epidemiologia7030087)

epidemiologia

Article

10-Year Change in the Laboratory-Based Prevalence of Chronic Kidney Disease in Patients from a Brazilian Cardiologic Center

Supplementary material

Table S1. Number and proportion of new patients with serum creatinine measurements each year.

Year	Without previous sCR measurement, % (*n*)	With previous sCR measurement, % (*n*)
2014	-	-
2015	33.2 (14,967)	66.8 (30,133)
2016	21.7 (8797)	78.3 (31,703)
2017	18.7 (7029)	81.3 (30,576)
2018	13.3 (2820)	86.7 (18,408)
2019	24.6 (8935)	75.4 (27,343)
2020	15.8 (4448)	84.2 (23,708)
2021	18.1 (6803)	81.9 (30,752)
2022	16.3 (6137)	83.7 (31,527)
2023	15.6 (5549)	84.4 (30,021)

Table S2. Incidence of CKD according to sex.

	Male (Number at risk =20,250)		Female (Number at risk =19,164)		All (Number at risk =39,414)	
Period (years)	Cumulative CKD (*n*)	Cumulative CKD incidence (%)	Cumulative CKD (*n*)	Cumulative CKD incidence (%)	Cumulative CKD (*n*)	Cumulative CKD incidence (%)
0.0-0.9	43	0.21	48	0.25	91	0.23
1.0-1.9	866	4.28	794	4.14	1660	4.21
2.0-2.9	1572	7.76	1549	8.08	3121	7.92
3.0-3.9	2154	10.64	2135	11.14	4289	10.88
4.0-4.9	2707	13.37	2696	14.07	5403	13.71
5.0-5.9	3160	15.60	3166	16.52	6326	16.05
6.0-6.9	3556	17.56	3614	18.86	7170	18.19
7.0-7.9	3921	19.36	4003	20.89	7924	20.10
8.0-8.9	4187	20.68	4266	22.26	8453	21.45
+9.0	4232	20.90	4315	22.52	8547	21.69

Table S3. Incidence of CKD according to age group.

	18-29 years (Number at risk=642)		30-44 years (Number at risk =2908)		45-59 years (Number at risk =12,487)		60-74 years (Number at risk =18,471)		≥75 years (Number at risk =4906)		All (Number at risk =39,414)	
Period (years)	Cumul. CKD (*n*)	Cumul. CKD (%)	Cumul. CKD (*n*)	Cumul. CKD (%)	Cumul. CKD (*n*)	Cumul. CKD (%)	Cumul. CKD (*n*)	Cumul. CKD (%)	Cumul. CKD (*n*)	Cumul. CKD (%)	Cumul. CKD (*n*)	Cumul. CKD (%)
0.0-0.9	0	0.00	3	0.10	19	0.15	48	0.26	21	0.43	91	0.23
1.0-1.9	2	0.31	27	0.93	298	2.39	881	4.77	452	9.21	1660	4.21
2.0-2.9	3	0.47	46	1.58	562	4.50	1698	9.19	812	16.55	3121	7.92
3.0-3.9	7	1.09	59	2.03	806	6.45	2357	12.76	1060	21.61	4289	10.88
4.0-4.9	9	1.40	69	2.37	1023	8.19	3021	16.36	1281	26.11	5403	13.71
5.0-5.9	10	1.56	86	2.96	1219	9.76	3559	19.27	1452	29.60	6326	16.05
6.0-6.9	11	1.71	108	3.71	1401	11.22	4084	22.11	1566	31.92	7170	18.19
7.0-7.9	13	2.02	123	4.23	1589	12.73	4525	24.50	1674	34.12	7924	20.10
8.0-8.9	15	2.34	140	4.81	1731	13.86	4825	26.12	1742	35.51	8453	21.45
+9.0	15	2.34	140	4.81	1769	14.17	4871	26.37	1752	35.71	8547	21.69

Table S4. Cumulative incidence of CKD according to the methodology adopted for defining new cases.

Methodology for estimating CKD incidence	Number at risk (*n*)	10-y CKD identified (*n*)	10-y cumulative CKD incidence (%)							
			All	Male	Female	18-29 y	30-44 y	45-59 y	60-74 y	≥ 75 y
≥2 measurements of eGFR >60 before to ≥1 measurements of eGFR ≤ 60	39,414	8547	21.7	20.9	22.5	2.3	4.8	14.2	26.4	35.7
≥2 measurements of eGFR >60 before to ≥2 measurements of eGFR ≤ 60	30,534	3619	11.9	11.2	12.6	1.6	2.8	8.6	16.2	21.2

epidemiologia

Figure S1. Trends in the prevalence of reduced eGFR (<60 ml/min/1.73 m²), excluding the year 2020 (COVID-19 pandemic). (a) Crude prevalence; (b) age- and sex-adjusted prevalence.

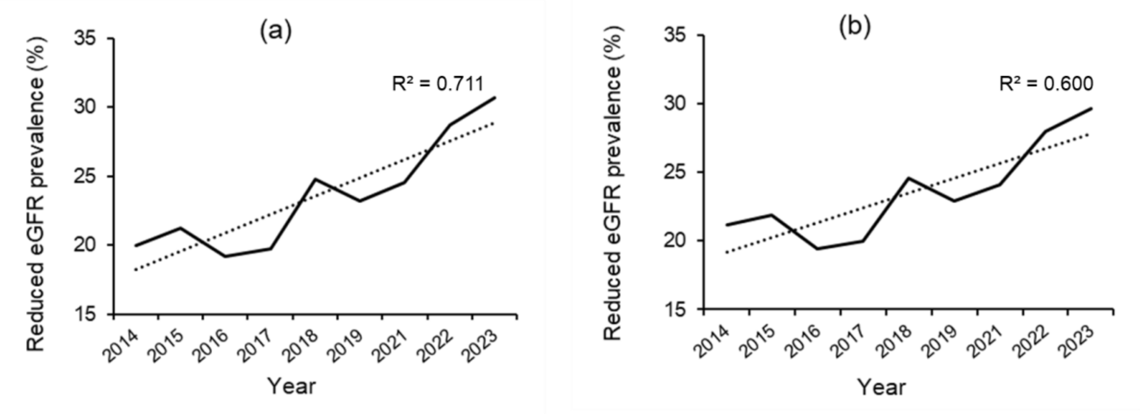

Supplement: Supplementary file 1 [file epidemiologia-07-00087-s001.zip › epidemiologia-4311931-supplementary.pdf]
